# The feedback loop of LITAF and BCL6 is involved in regulating apoptosis in B cell non-Hodgkin's-lymphoma

**DOI:** 10.18632/oncotarget.12680

**Published:** 2016-10-15

**Authors:** Yaoyao Shi, Yue Kuai, Lizhen Lei, Yuanyuan Weng, Friederike Berberich-Siebelt, Xinxia Zhang, Jinjie Wang, Yuan Zhou, Xin Jiang, Guoping Ren, Hongyang Pan, Zhengrong Mao, Ren Zhou

**Affiliations:** ^1^ Department of Pathology and Pathophysiology, Institute of Pathology and Forensic Medicine, Zhejiang University School of Medicine, Hangzhou, China; ^2^ Department of Pathology, Sir Run Run Shaw Hospital, Zhejiang University School of Medicine, Hangzhou, China; ^3^ Institute of Pathology, Wuerzburg University, Wuerzburg, Germany; ^4^ Epitomics Inc., Hangzhou, China; ^5^ Department of Pathology, Hangzhou First People's Hospital, Hangzhou, China; ^6^ Postgraduate School in Medical School of Ningbo University, Ningbo, China; ^7^ Department of Pathology, the First Affiliated Hospital, Zhejiang University School of Medicine, Hangzhou, China

**Keywords:** LITAF, BCL6, transcription, apoptosis, B-NHL

## Abstract

Dysregulation of the apoptotic pathway is widely recognized as a key step in lymphomagenesis. Notably, *LITAF* was initially identified as a p53-inducible gene, subsequently implicated as a tumor suppressor. Our previous study also showed *LITAF* to be methylated in 89.5% B-NHL samples. Conversely, deregulated expression of BCL6 is a pathogenic event in many lymphomas. Interestingly, our study found an oppositional expression of LITAF and BCL6 in B-NHL. In addition, *LITAF* was recently identified as a novel target gene of BCL6. Therefore, we sought to explore the feedback loop between LITAF and BCL6 in B-NHL. Here, our data for the first time show that LITAF can repress expression of *BCL6* by binding to Region A (−87 to +65) containing a putative LITAF-binding motif (CTCCC) within the *BCL6* promoter. Furthermore, the regulation of BCL6 targets (*PRDM1* or *c-Myc*) by LITAF may be associated with B-cell differentiation. Results also demonstrate that ectopic expression of LITAF induces cell apoptosis, activated by releasing cytochrome c, cleaving PARP and caspase 3 in B-NHL cells whereas knockdown of LITAF robustly protected cells from apoptosis. Interestingly, BCL6, in turn, could reverse cell apoptosis mediated by LITAF. Collectively, our findings provide a novel apoptotic regulatory pathway in which LITAF, as a transcription factor, inhibits the expression of *BCL6*, which leads to activation of the intrinsic mitochondrial pathway and tumor apoptosis. Our study is expected to provide a possible biomarker as well as a target for clinical therapies to promote tumor cell apoptosis.

## INTRODUCTION

*LITAF*, encoding a transcription factor, was initially identified as the p53- inducible gene7 (termed *Pig7*) in 1997 [1]. Subsequent studies have demonstrated that LITAF could bind to a sequence motif, CTCCC (−515 to −511), within the *TNF* promoter, activating transcription of *TNF* upon lipopolysaccharide (LPS) stimulation [2, 3]. Apart from its critical role in inflammatory response, mutations in *LITAF* are associated with Paget's disease [4] and Charcot-Marie-Tooth disease (CMT) [5, 6]. Recently, accumulating evidences have indicated that LITAF may be considered as a tumor suppressor in different malignancies. For instance, Zhou et al. previously characterized LITAF as one of downstream targets of AMPK to inhibit cancer cell growth, through up-regulation of TNFSF15 in prostate cancer cells [7]. LITAF was also identified to promote cell apoptosis and differentiation in acute myeloid leukaemia [8], and the decreased expression was observed in breast cancer [9].

In mature B-cell lymphoma, *LITAF* is inactivated by epigenetic mechanisms [10], but the biological functions of LITAF remain to be discovered. Recent studies have showed, both mRNA and protein expression of LITAF are significantly down-regulated in germinal centre (GC) B-cell-like diffuse large B-cell lymphoma (GCB-DLBCL), which display constitutively high BCL6 expression [11–13]. Interestingly, it has been demonstrated that *LITAF* is a novel target of BCL6 [14], providing new insights into the crosstalk between BCL6 and LITAF. In fact, BCL6 is a transcriptional repressor of the POZ/BTB zinc-finger protein family, acting as a key regulator for development of the germinal center (GC) and further differentiation [15]. Of note, BCL6 is required to sustain proliferation and survival of DLBCL cells through regulation of specific targets such as *PRDM1*, *c-Myc* or *PAX-5* [16–18]. Additionally, the role of BCL6 in preventing cell apoptosis is demonstrated by previous studies about its repression of *p53* [19, 20], *PDCD2* [21–22] or caspase 3 cascade [23] and its cooperation with Bcl-XL [24]. Accordingly, to improve understanding about the functions of LITAF in B-NHL, we focus on the negative correlation between BCL6 and LITAF. We propose the hypothesis that an apoptosis pathway is induced by LITAF, and partly reversed by BCL6.

The fact that B-NHL is a highly heterogeneous disease with different biology and clinical outcome, prompt current studies to find a potential biomarker for clinical diagnosis or therapy. Our primary study showed *LITAF* to be silenced due to aberrant CpG methylation in B-NHL cases, which is probably a critical event for the oncogenesis of B-NHL. In this study, we offer an explanation for the anti-oncogenic function of LITAF, which may act as a novel proapoptotic activator through the intrinsic mitochondrial pathway. Furthermore, LITAF might facilitate cell apoptosis and terminal differentiation by transcriptional repression of *BCL6*, thereby increasing the likelihood of other genetic events associated with the development of B-cell lymphomas. In sum, our work provides novel insights into the properties of LITAF-associated BCL6 repression and induction of apoptosis, with broader implications for therapy development by targeting cell apoptosis in B-NHL.

## RESULTS

### Expression of LITAF correlates inversely with BCL6 in B-NHL

**Figure 1:**
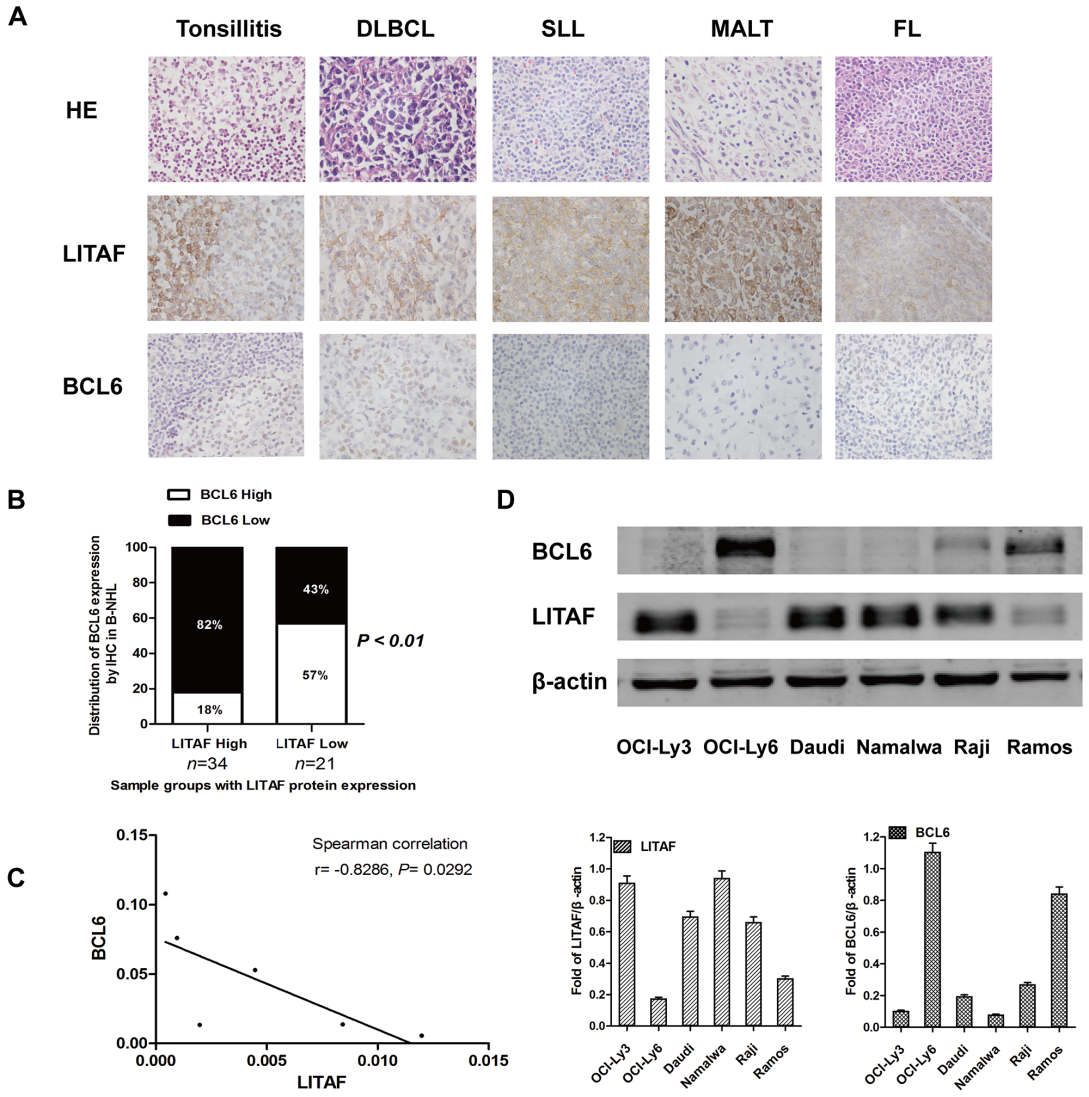
LITAF inversely correlates with BCL6 in B-NHL **A.** Representative histopathological morphology and immunohistochemical images of reactive human tonsil, DLBCL, SLL, MALT or FL staining for LITAF and BCL6 (×400 objective magnifications). **B.** The quantification about IHC of B-NHL samples for LITAF and BCL6 analysis (n = 55; *P* = 0.0024, χ^2^ test). **C.** The mRNA levels of *LITAF* and *BCL6* in 6 B-NHL cell lines were evaluated by Spearman's rank correlation (r = −0.8286; *P* = 0.0292). **D.** Western blotting and quantification for LITAF and BCL6 in six B-NHL cell lines, the relative expression values were normalized to β-actin. At least three independent experiments were carried out.

To explore the biological functions of LITAF in B cells, we detected the expression of LITAF and BCL6 in 55 B-NHL samples by immunochemistry (Table 1). The data showed cytoplasmic expression of LITAF surrounding GCs, whereas the expression of BCL6 was located within GC cells in reactive tonsils (Figure 1A). Our findings about different subtypes of B-NHL cases with negative correlation between BCL6 and LITAF (Supplementary Table S1, Figure 1A and 1B) were consistent with a previous study. Here, the inverse association between the two proteins was also significant in DLBCLs (data not show), which may offer a possibility for further investigation. In addition, the expression of LITAF and BCL6 was assayed in six B-NHL cell lines including Burkitt's lymphoma cell lines (Raji, Daudi, Ramos and Namalwa) and DLBCL cell lines (OCI-Ly3 and OCI-Ly6) at mRNA and protein level. In accordance with the IHC data, a strong linear correlation between *LITAF* and *BCL6* mRNA expression levels was observed in B-NHL cells evaluated by Spearman's correlation (r = −0.8286; *P* = 0.0292) (Figure 1C). As shown in Figure 1D, all cell lines expressed LITAF, although at variable levels. Cells displaying the lowest expression levels between the two B-NHL types were then selected for further analysis (Ramos and OCI-Ly6, respectively). OCI-Ly3 and Namalwa cell lines were also used as experimental models due to their highest expression of LITAF. Collectively, these results establish a relevant inverse relationship between LITAF and BCL6, implicating the functions of LITAF to be associated with the crosstalk with BCL6 in B-NHL cells.

**Table 1:** Distribution of LITAF and BCL6 staining in 55 B-NHL cases

	cases	LITAF protein		BCL6 protein	
		low	high	low	high
DLBCL	36	13	23	22	14
SLL	8	5	3	7	1
MALT	4	0	4	4	0
FL	3	2	1	1	2
Burkitt's	1	0	1	1	0
Undetermined	3	1	2	2	1

Abbreviations: DLBCL, diffuse large B cell lymphoma; SLL, small B cell lymphoma; MALT, mucosa-associated lymphoid tissue; FL, follicular lymphoma; Burkitt's, Burkitt's lymphoma.

### The transcription factor LITAF directly represses *BCL6* as a feedback

**Figure 2:**
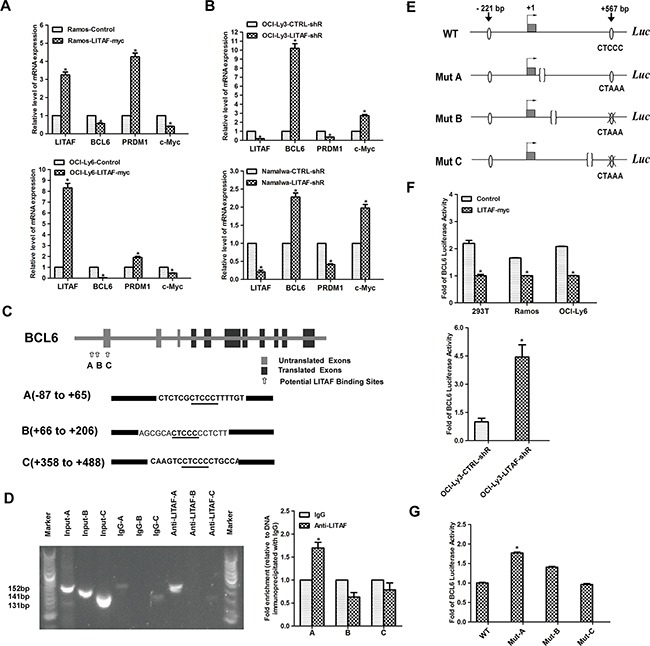
Transcriptional regulation of *BCL6* by LITAF **A.** qRT-PCR showing temporal regulation of *BCL6* and its target genes by LITAF in Ramos and OCI-Ly6 cells 48 h post-infection. Control, cells with pLVX virus; LITAF-myc, cells with over-expressed LITAF. **B.** Effect of silencing LITAF on *BCL6*, *PRDM1* and *c-Myc* mRNA in OCI-Ly3 and Namalwa cells. LITAF-shR, shRNA against LITAF; CTRL-shR, scrambled shRNA. **C.** Graphic representation of the *BCL6* gene highlighting the three potential LITAF binding sites. The sequences of A (−87 to +65), B (+66 to +206) and C (+358 to +488) of the *BCL6* gene are shown containing the LITAF consensus sites (CTCCC) underlined. Sequence numbers are in reference to the + 1 nucleotide identified by previous study [43]. **D.** ChIP assay in OCI-Ly3 cells. PCR was performed with primers specific for three potential regulating regions (A, B and C) in the promoter of *BCL6*, respectively. The PCR product was analyzed by agarose gel electrophoresis (left), and qRT-PCR results are expressed as fold enrichment calculated as the percentage of input for the specific antibody (LITAF) with respect to IgG control of three replicates (right). Marker, 100-bp ladder. **E.** Schematic representation of the wild-type and three mutant (CTCCC to CTAAA) *BCL6* reporters used in the experiments. **F.** The luciferase activity of *BCL6* reporter plasmid in cell lines including 293T, Ramos and OCI-Ly6 after over-expression of LITAF (upper); and OCI-Ly3 cells infected with shRNA for LITAF or control virus (lower). **G.** Luciferase activity of wild-type (WT) or mutant (Mut-A, Mut-B and Mut-C) *BCL6* reporters in OCI-Ly6 cells transfected with LITAF. Mean±s.d. of three technical replicates were plotted. *P < 0.05, Student's *t*-test.

A recent study for the first time has demonstrated that *LITAF*, a novel target of BCL6, regulates autophagy in mature B-cell lymphomas [14]. This study provided evidences for the transcriptional repression of *LITAF* by BCL6 in B cells. Here, we found that there may be a reciprocal feedback loop between LITAF and BCL6. Interestingly, LITAF could regulate *BCL6* expression at mRNA level. Compared with the control groups, the expression of *BCL6* was significantly decreased in Ramos and OCI-Ly6 cells after over-expression of LITAF (Figure 2A). In contrast, *BCL6* was up-regulated after silenced LITAF in OCI-Ly3 and Namalwa cell lines (Figure 2B). These data indicated that LITAF could regulate the expression of *BCL6* as a feedback, which aroused our interest. We next determined if this led to changes in BCL6 transcriptional activity. Therefore, we analysed the expression of BCL6 target genes *(PRDM1 and c-Myc*) to determine if modulation of *BCL6* mRNA expression by LITAF resulted in changes in its targets. We found that *PRDM1* was significantly increased along with down-regulated *c-Myc* in Ramos and OCI-Ly6 cells (Figure 2A). As we expected, for OCI-Ly3 and Namalwa cells, knockdown of LITAF decreased *PRDM1* and up-regulated *c-Myc* (Figure 2B). Therefore, the activation status of LITAF has direct effects on BCL6 functions, as measured by the expression changes of BCL6 target genes.

To investigate the underlying mechanism about the repression of *BCL6*, ChIP assays were performed to determine whether LITAF was able to bind to the *BCL6* promoter. Considering that mutations in 5' noncoding sequences of *BCL6* were frequent in B-cell lymphoma, which might contain potential regulatory regions [25], three pairs of primers were designed to amplify enrichment sites with specific binding motif (CTCCC) around the first exon of *BCL6*, respectively (Figure 2C) [26]. Following ChIP of OCI-Ly3 cells with an antibody specific for LITAF, the fragment of 152 bp containing the putative LITAF binding site at Region A was successfully amplified (Figure 2D). Additionally, similar results were showed in qRT-PCR analysis (Figure 2D). These data indicated that *in vivo* LITAF was binding to Region A (−87 to +65)of *BCL6* gene. To further validate the predicted target, we performed the luciferase reporter assays. Compared with control cells, luciferase activities decreased significantly in Ramos and OCI-Ly6 cells with over-expressed LITAF (Figure 2F). Conversely, the BCL6 luciferase activity in LITAF-silenced OCI-Ly3 cells increased significantly in comparison to that in control cells (Figure 2F). To assess whether the previously documented phenotypic effects were due to the specific binding motif (CTCCC) in Region A, Ramos-LITAF cells were transfected with *BCL6* wild-type reporter, or three mutant *BCL6* reporters containing motif (CTCCC to CTAAA) (Figure 2E). Consistent with ChIP assay data (Figure 2D), LITAF sensitivity of BCL6 required an intact Region A and therefore, the responsive element to be in its appropriate genomic context (Figure 2G). Taken together, LITAF can function as a transcription factor, associated with transcriptional repression of *BCL6*.

### LITAF interacts with BCL6 *in vivo*

**Figure 3:**
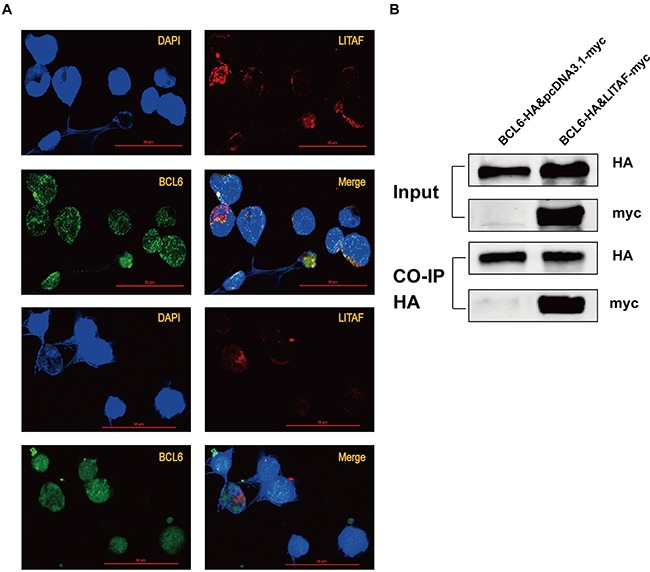
The location and interaction of LITAF and BCL6 protein **A.** Detection by immunofluorescence of LITAF (red), BCL6 (green) and DAPI (blue) in OCI-Ly6 cells transfected with LITAF-myc. Representative images showing localizations of LITAF and BCL6 from replicate experiments. Bar, 50μm. **B.** Co-IP was used to analyze the binding ability between LITAF (with myc tag) and BCL6 (with HA tag) in 293T cells. Each experiment was performed independently at least three times.

Studies previously identified LITAF as a transcription factor, activating transcription of *TNF* upon LPS stimulation [2, 3]. Under basal conditions, LITAF was expressed in the cytoplasm. However, LITAF translocated into the nucleus and modulate expression of inflammatory cytokines upon activation [27]. Accordingly, we performed immunofluorescent assays to confirm the hypothesis that LITAF, as a transcription factor, would translocate to the nucleus and further regulate its target genes. We used double IF on fixed LITAF-over-expressing OCI-Ly6 cells, consecutively stained with anti-LITAF-Alexa-594 and anti-BCL6-Alexa-488 antibodies. As the data showed in Figure 3A (replicate experiments), continuous expression of LITAF could be found in the nucleus, supporting the transcriptional regulation of *BCL6*. Furthermore, we found that co-transfection of LITAF and BCL6 in 293T cells induced an interaction (Figure 3B), which reveal the existence of a protein-protein interaction between the two transcription factors *in vivo.*

### LITAF positively regulates apoptosis through intrinsic mitochondrial pathway in B-NHL cell lines

**Figure 4:**
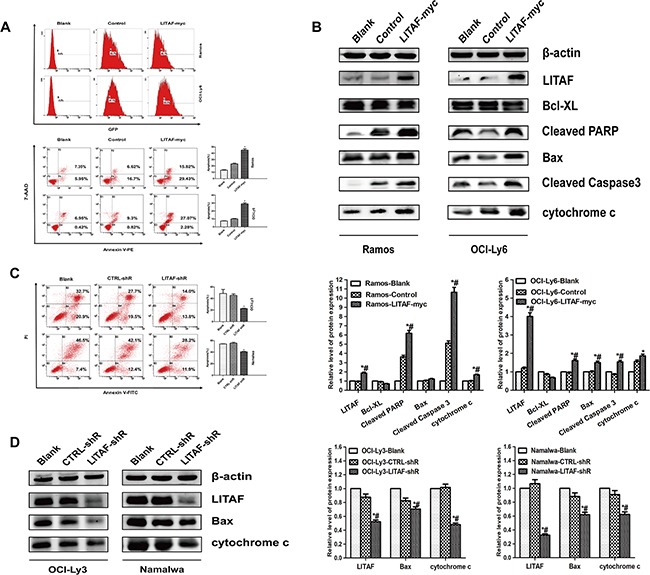
The function of LITAF involved in regulating apoptosis in B-NHL cells **A.** Apoptosis assay for Ramos (upper panel) and OCI-Ly6 cells (lower panel) infected with either LITAF-myc or control virus (pLVX) along with blank (non-infected cells), quantification analysis was shown on the right. *P < 0.05, LITAF-myc versus control, while #*P* < 0.05, LITAF-myc versus blank. **B.** Immunoblotting for LITAF, Bcl-XL, Cleaved PARP, Bax, Cleaved Caspase 3 and cytochrome c in Ramos and OCI-Ly6 cells with over-expressed LITAF or control (upper panel). β-actin was used as loading control. The quantitative analysis for western blot was reported from three independent experiments (lower panel). **C.** Apoptosis analysis (left) and quantification (right) for the effect of silenced-LITAF in OCI-Ly3 and Namalwa cell lines. *P < 0.05, LITAF-shR versus CTRL-shR, while #*P* < 0.05, LITAF-shR versus blank. **D.** Western blotting and quantification of LITAF, Bax, cytochrome c and β-actin were analyzed in blank cells and the stably-infected lymphoma cell lines including CTRL-shR cells and LITAF-shR cells. The result shown is a representative of three independent experiments.

With regard to LITAF that was dramatically up-regulated upon activation of p53 [1], we hypothesized that LITAF may activate apoptosis in B-NHL cells. As shown in Figure 4A, compared with the control groups (Blank and Control), LITAF overexpression in Ramos and OCI-Ly6 cells could particularly induce apoptosis. In line with LITAF-supported apoptotic events, a rise in Bax and fall in Bcl-XL (although not significant), release of cytochrome c, as well as cleavage of PARP and caspase 3 were observed (Figure 4B). Simultaneously, LITAF was knockdown in OCI-Ly3 and Namalwa cells, and apoptosis was assessed after treatment with hydrogen peroxide combined with serum deprivation. Interestingly, LITAF-silenced OCI-Ly3 and Namalwa cells displayed a decrease in apoptosis along with significant repression of Bax expression and cytochrome c release (Figure 4C and Figure 4D). Thus, these results above suggest that LITAF may act as a proapoptotic activator and induce cell apoptosis through intrinsic pathway in B-cell lymphoma.

### BCL6 inhibits the apoptosis activated by LITAF in B-NHL cell lines

**Figure 5:**
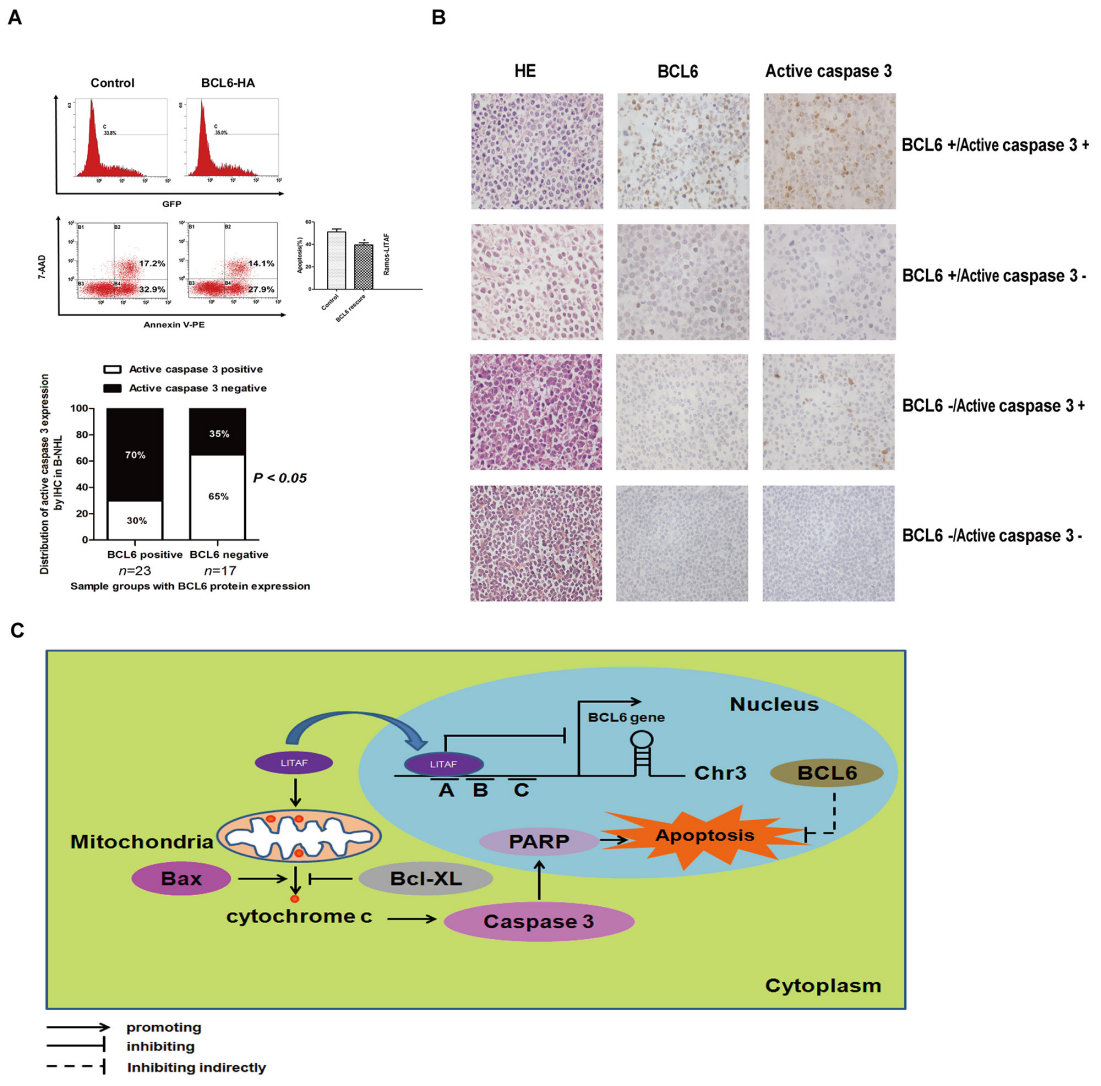
BCL6 inhibit cell apoptosis mediated by LITAF in B-NHL **A.** Compared with control, partial recovery of LITAF induced apoptosis by the over-expression of BCL6. Ramos-LITAF, Ramos cell lines transfected with LITAF; the right column, quantitative analysis of apoptosis, with statistics. **B.** IHC and quantification for BCL6 and active caspase 3 in B-NHL cases (n = 40; *P* = 0.0313, χ2 test). Each experiment was performed at least three times. *P < 0.05. **C.** Pathway of LITAF induction of apoptosis. In the nucleus, BCL6 was repressed by transcription factor LITAF by binding the region A. The overexpressed LITAF in cytoplasm could activate the intrinsic apoptosis pathway by releasing cytochrome c from mitochondria, cleaving caspase 3 and PARP. The upregulation of BCL6 could reverse cell apoptosis as a feedback.

Our previous results revealed a cross-talk between LITAF and BCL6. In order to further investigate whether BCL6 was involved in the same pathway of apoptosis, we electro-tansfected a BCL6 expression vector or control plasmid into LITAF over-expressing Ramos cells. As the data showed, over-expressed BCL6 could partly reverse the apoptosis rate (Figure 5A), indicating that there was a balance between BCL6 and LITAF to determine the fate of cells. Moreover, we analyzed the distribution of BCL6 and active caspase 3 expression in 40 B-NHL samples. Interestingly, a negative correlation between the two proteins was observed (Figure 5B), suggesting that BCL6 may function to repress apoptosis in B-NHL. The fact that the documented repression of *p*53 [19, 20], *PDCD2* [21, 22] or the caspase 3 cascade [23] by BCL6 may offer an explanation for the anti-apoptotic role of BCL6. Overall, these data indicate that *BCL6*, as an oncogene involved in lymphomagenesis, can reverse cell apoptosis induced by LITAF.

## DISCUSSION

In our study, we have for the first time identified *BCL6* as a possible novel target of LITAF. Accordingly, LITAF translocated into the nucleus where it could bind to the Region A of the *BCL6* promoter. This leads to downregulation of *BCL6* expression as well as changes in BCL6 target genes related to B-cell differentiation. In addition, our data show an *in vivo* protein interaction between LITAF and BCL6, hinting towards a combined mechanism. The inhibition of *BCL6* by LITAF is not only via transcriptional repression, but also partly via protein–protein interaction. Further results prove that ectopical expression of LITAF can induce cell apoptosis through an intrinsic mitochondrial pathway, and the repression of BCL6 may be responsible for apoptosis activated by LITAF (Figure 5C).

LITAF, an activator of *TNF* upon LPS stimulation, functions as a transcription factor in the inflammatory response [3]. In line, Tang et al. previously demonstrated a novel LITAF-binding site (CTCCC) on the *TNF* promoter [2]. Besides that, LITAF inhibits cell proliferation by specially binding to the same consensus motif, CTCCC (−515 to −511), and activating transcription of *TNFSF15* in prostate cancer [7]. Differences may rely on the network of transcription factors. For example, a functional interaction between LITAF and STAT6 (B) has been identified in monocytes and macrophages, which is important for the expression of several cytokines [27]. Nevertheless, studies above support that LITAF acts as a transcription factor involved in the regulation of pro-inflammatory cytokines or genes relevant to tumorigenesis. Surprisingly, LITAF did not induce TNF secretion upon LPS stimulation in B-cell lymphomas, and subcellular localization determined LITAF to be cytoplasmic only [14]. On the contrary, we characterized LITAF as a transcription factor in B-cell lymphoma, translocating to the nucleus and repressing *BCL6* mRNA expression by binding to its regulatory region (Figure 2C-2G and Figure 3A). In addition, the detected protein-protein interaction between LITAF and BCL6 might counteract BCL6 activity (Figure 3B). Intriguingly, there is a prerequisite for shielding BCL6 activity by another transcription factor, namely T-bet, which masks the *BCL6* DNA-binding domain in T-helper 1 cells [28]. LITAF on the other hand might depend on interacting transcription factors to translocate to the nucleus as revealed for the LITAF-STAT6 (B) complex [27]. Thus, if interaction with BCL6 enables nuclear translocation of LITAF, which subsequently represses *BCL6* expression, this could explain the observed differences in subcellular localization between the two reports on B-cell lymphoma.

As the proto-oncogene *BCL6* is a master regulator of B-lymphocyte development, it inhibits expression of *PRDM1* and blocks further differentiation [16, 29]. *c-Myc* is repressed by PRDM1, which explains cessation of cell cycle in plasma cells as it is required for proliferation and growth [17, 30, 31]. Consistently, LITAF-associated inhibition of BCL6 relieved the repression of *PRDM1* along with downregulation of *c-Myc* (Figure 2A). On the other hand, knock-down of LITAF could up-regulate expression of *BCL6* and force repression of *PRDM1*, whereas *c-Myc* expression subsequently increased (Figure 2B). Therefore, the repression of *BCL6* by LITAF and changes in its targets implicate that LITAF may indirectly contribute to plasma cell differentiation. Nonetheless, a detailed examination of physiological signals, which regulate LITAF expression in B- or plasma cells, is missing. At least in B-cell lymphoma, LPS rarely induces LITAF [14] and the LITAF-regulated pathway in B-NHL cells might thus yield additional experiments.

Notably, a possible cross-talk between LITAF and BCL6 has been supported by the recent findings that *LITAF* is a novel target of BCL6 [14]. The report showed about the negatively correlated expression level between LITAF and BCL6 was coincidently consistent with our data (Figure 1). Further experiments pointed to a role of LITAF in regulating apoptosis in B cells (Figure 4A and 4C), suggesting a link between BCL6 and apoptosis. Given that LITAF was initially identified as a target in p53-induced apoptosis [1] and interaction between CIDE-3 and LITAF might be involved in the apoptosis of liver cells [32]. In line, differentially regulated players of the intrinsic mitochondrial pathway (Figure 4B and 4D) imply the possible apoptotic mechanism mediated by LITAF. Interestingly, BCL6 overexpression can immortalize p53-deficient B cells [33], or protect cells from apoptosis by repressing p53 [19]. Furthermore, BCL6 may act as a stabilizer in protecting spermatocytes from apoptosis induced by stressors [23] and enhance the viability of differentiating myocytes by preventing apoptosis [34]. In agreement, *PDCD2* is a target of BCL6 repression [21, 22]. Accumulating evidences indicate that BCL6 is a positive regulator of B-cell proliferation, which could remain cell viability by inhibiting apoptosis. Importantly, these reports were consistent with our findings where we could place *BCL6* as an oncogene, which partly reverses cell apoptosis activated by LITAF (Figure 5A). Additionally, apoptosis is repressed significantly by the caspase 3-directed peptide inhibitor in the BCL6-knock-down cells [23]. In our work, the inverse correlation between BCL6 and active caspase 3 in 40 B-NHL cases (Figure 5B), implies that apoptosis reversed by BCL6 may be relative with the repression of caspase 3 cascade. Thus, it is likely that the crosstalk mediates specifically the LITAF-dependent BCL6 repression and subsequent induction of cell apoptosis by the activation of intrinsic mitochondrial pathway.

Our primary study has demonstrated that *LITAF* is methylated with high frequency in B-NHL cases, probably a critical event to the lymphomagenesis. Interestingly, the follow-up study has also showed 57% (4/7) percent of cases with poor survival displaying lower expressed LITAF (data not shown), which needs to expand further samples. Studies above implicate that LITAF, as a positive regulator of apoptosis like p53, might act as a similar tumor suppressor suggested by other groups [7, 9, 14]. It has been reported that the repression of *LITAF* by BCL6 is essential to ensure a rapidly proliferating state of those B cells in GCB-DLBCL [14]. As apoptosis is involved in the physiological development of B cells within the GCs [35, 36], the feedback loop between BCL6 and LITAF may be involved in regulating apoptosis during the somatic hypermutation and proliferation processes. On the contrary, continuous expression of anti-apoptotic proteins or mutations in apoptosis-inducing genes play a critical role in lymphomagenesis [37]. Therefore, understanding the fine tuning between apoptosis and lymphomagenesis as exemplified by the presented feedback loop between BCL6 and LITAF may offer hope for cancer diagnosis and therapy.

In conclusion, our study for the first time identifies *BCL6* as a novel target of LITAF, which is involved in regulating cell apoptosis in B-NHL. Our work may also provide a new apoptotic pathway, in which ectopic LITAF induces apoptosis by activating caspase cascade which could be reversed by BCL6 as a feedback. Consequently, further experiments need to be clarified whether LITAF may function as a promising target with therapeutic value in B cell lymphoma.

## MATERIALS AND METHODS

### Cell cultures

293T and Burkitt's lymphoma cell lines (Raji, Daudi and Namalwa) were obtained from the Type Culture Collection of the Chinese Academy of Sciences (Shanghai, China); Ramos (Burkitt's lymphoma cell line) cell line was purchased from the ATCC; DLBCL cell lines OCI-Ly3 and OCI-Ly6 were kindly provided by Dr. X. Jiang (China) and Professor T. Zhao (China), respectively. The 293T cells were maintained in DMEM supplemented with 10% FBS (Gibco). Burkitt's lymphoma cell lines were cultured in RPMI-1640 with 10% FBS (Gibco), and the DLBCL cell lines were maintained in IMDM with 10% FBS (Gibco). Cultures were maintained in a 5% CO_2_ humidified atmosphere at 37 °C.

### DNA constructs

The LITAF gene was PCR-amplified from 293T cDNA and sub-cloned with a six-repeating myc tag at its amino terminus into the pLVX-IRES-ZsGreen1 plasmid (Clontech Laboratories), named LITAF-myc-pLVX-IRES-ZsGreen1 (LITAF-myc). A set of three LITAF siRNA were transfected into 293T cells to examine a clone that produces the most efficient silencing effect. Double-stranded oligonucleotides coding for siRNA that specifically target LITAF transcript (sense: 5′-GUGUCAUCUUUG- AAGUCAAGA-3′ and anti-sense: 5′-UUGACUUCAAAGAUGACACAG-3′) were cloned into the pLKO.1 vector (Addgene) by Integrated Biotech Solutions (Shanghai, China). The plasmid was sequenced and named pLKO.1-puro-LITAF-shRNA (LITAF-shRNA). PLKO.1-puro control plasmid (CTRL-shRNA) contained an insert with non-target. *BCL6* promoter construct was created by amplifying −211 to +567 fragments using BCL6-Luc primers (Supplementary Table S3) and inserting them upstream of the SV40 promoter in the luciferase pGL3 vector (Promega) [38]. Additionally, we constructed three mutant *BCL6* reporters (CTCCC to CTAAA) using primers listed in Supplementary Table S3, termed BCL6-Mut-A, BCL6-Mut-B and BCL6-Mut-C, respectively [39].

### LITAF gain and loss of function experiments

Lentivirus was packaged by different recombinant plasmids along with helper plasmids (psPAX2 and pMD2.G) in 293T cells, and virus supernatants were collected at 48 h and 72 h post-transfection. After concentration, recombinant LITAF-myc- pLVX-IRES-ZsGreen1 virus or control (pLVX-IRES-ZsGreen1) virus was infected into OCI-Ly6 and Ramos cells. Additionally, LITAF was silenced with recombinant LITAF-pLKO.1-puro virus in OCI-Ly3 and Namalwa cells, and control cells were generated using a non-target scramble. After infection, stable clones with shRNA were selected with puromycin (Invitrogen) at a final concentration of 2μg/ml.

### Quantitative real time PCR (qRT-PCR)

Total RNA was isolated from cell lines by Trizol (Invitrogen) according to the manufacturer's protocol, and reverse-transcribed using a PrimeScript RT reagent kit (Takara). Real-time PCR was performed three times in triplicate with SYBR Premix Ex TaqTM (Takara), using the CFX96 Touch™ Real-Time PCR System (BIO-RAD). The real-time primer sequences were listed in Supplementary Table S3. Relative expression levels was normalized to GAPDH and calculated with the 2^−ΔΔct^ method. Each experiment was performed independently at least three times.

### Chromatin immunoprecipitation (ChIP) assays

ChIP assays were performed according to the protocol of EZ-ChIP kit (Merck Millipore). OCI-Ly3 cells were cross-linked with formaldehyde (1% final concentration), quenched and lysed in lysis buffer. 4μg of antibody for LITAF (sc-166546, Santa Cruz) or normal mouse IgG was applied to precipitate chromatin from 2×10^6^ cells. Primers were designed to amplify three regions containing a specific binding site (CTCCC) each for LITAF: Region A (nucleotides −87 to +65); Region B (nucleotides +66 to +206); and Region C (nucleotides +358 to +488) with primers shown in Supplementary Table S3. Immunoprecipitated DNA and input samples were analyzed by agarose gel electrophoresis and qRT-PCR as described above. ChIP values were normalized to input and expressed as mean fold change relative to nonspecific IgG of three replicates.

### Luciferase reporter assay

Cells were plated in 12-well plates at a density of 1×10^6^ cells/well. For luciferase assays in Ramos and OCI-Ly6 cells, 1μg of LITAF-pCDNA3.1(+)6myc or pCDNA3.1(+)6myc, together with 1μg of BCL6 promoter constructs, and 20 ng of pRL-TK were transiently transfected into the cells by electro-transfection (Nucleofector II, Amaxa Biosystems) using the Amaxa Cell Line Nucleofector Kit V (Lonza). For stably LITAF-silenced Namalwa and OCI-Ly3 cells or control cells, 250 ng of BCL6 reporter plasmids, and 10 ng of pRL-TK were transiently transfected by Lipofectamine™ 3000 (Invitrogen). Despite the wild-type BCL6 reporter assay, we mutated the specific motif (CTCCC to CTAAA) in three different sites of the promoter. OCI-Ly6 cells were transfected in duplicate with 1μg of LITAF- pCDNA3.1(+)6myc, together with 1μg of plasmids encoding either wt or mutant forms of *BCL6* reporters and pRL-TK. After 24-36 h, cells were lysed and luciferase activities were measured by the Dual-Luciferase Reporter Assay System (Promega). Three replicates were performed for each condition, and at least three independent experiments were carried out.

### Western blotting

The protein samples were resolved by SDS-polyacrylamide gel electrophoresis (PAGE), electroph oretically transferred to PVDF membranes (Millipore) and blocked in 5% BSA or nonfat milk in PBS/Tween-20. Antibodies specific for BCL6, Cleaved-PARP, Cleaved-caspase 3, Bcl-XL, Bax and cytochrome c were purchased from Cell Signaling Technology. The anti-LITAF, anti-actin, anti-HA and anti-c-Myc antibodies were purchased from Santa Cruz Biotechnology. The experiments for the immunoblotting were performed at least three times.

### Apoptosis assay

Ramos and OCI-Ly6 cells were infected with control or LITAF-pLVX-IRES-ZsGreen1 virus for 48-96h. 2×10^5^ cells were harvested with ice-cold phosphate buffered saline (PBS). In addition, Ramos cells with up-regulated LITAF were transfected with BCL6-pCDNA3.1(−)2HA or control vector. Cells were also collected and stained with Annexin V-PE along with 7-AAD, then infection efficiency (percentage of GFP) and apoptosis assay were detected by flow cytometry. LITAF-silenced Namalwa and OCI-Ly3 cells or control cells, treated with hydrogen peroxide combined serum deprivation, were assayed by AnnexinV-FITC and propidium iodide staining. The quantitative analysis of the percentage of positive cells was reported from three independent experiments.

### Co-Immunoprecipitation (Co-IP)

293T cells were co-transfected with BCL6 (with HA tag) and LITAF vectors (with myc tag) by Lipofectamine 3000 (Invitrogen). 36 h after transfection, the harvested cells were lysed on ice for 1 h using RIPA buffer (P0013, Beyotime) with 1 mM phenylmethanesulfonyl fluoride. Co-IP was performed as described previously [40]. Briefly, 2 ul anti-HA antibody (sc-7392, Santa Cruz) was added to the whole-cell lysate. The immune complexes were incubated at 4°C with rotation and collected using 10 ul protein G Plus-Agarose Immuno-precipitation Reagent (sc-2002, Santa Cruz). After that, the collected agarose beads were boiled in SDS sample and analyzed by western blotting. All the experiments were performed in triplicate.

### Immunohistochemistry

A total of 55 B-NHLs paraffin-embedded samples (Table 1) were obtained from the First Affiliated Hospital of Zhejiang University, and Department of Pathology and Pathophysiology of Zhejiang University after necessary informed. In each case, diagnosis of B-NHL was made according to the World Health Organiza- tion Classification of Tumors of Hematopoietic and Lymphoid Tissues [41]. This study was approved by the Ethics Committee of Zhejiang University (Hangzhou, China). IHC staining was performed using the EnVision system (Maixin Biotech) with antibodies for BCL6 (1:500, Dako), LITAF (1:2000, Abcam) and active caspase 3(1:400, Abcam). Data were calculated as previously described [42], and all scores were subdivided into two groups: low expression (≤median) and high expression (>median).

### Immunofluorescence staining

After 24 h transfection, OCI-Ly6 cells were washed twice in PBS, cytospun onto slide, air dried and fixed in acetone at −20 °C for 15 min. After treatment of 0.3% Triton X-100 (KeyGen Biotech) for 10 min, the slides were blocked with 10% BSA for 1 h at room temperature. The cells were then incubated with the primary antibody against LITAF (diluted 1:200, Santa Cruz) and BCL6 (diluted 1:200, CST) overnight at 4°C. Signals were detected with Alexa Fluor 488 and Alexa Fluor 594, respectively. Nuclei in live cells were stained with 40, 6-diamidino-2-phenylindole (DAPI), and visualized with laser confocal microscopy (Zeiss).

### Statistical analysis

Differences in immunoexpression frequencies were assessed using the Chi-square test. For *in vitro* and *in vivo* assays, data was analyzed using Student's t-test to compare the results among experimental groups and control groups. Statistical analysis was carried out using Graph Pad Prism version 5. Significance level was set at *P* < 0.05.

## SUPPLEMENTARY MATERIALS TABLES





## Abbreviations

LITAF: Lipopolysaccharide-induced TNF-alpha factor
BCL6: B-cell CLL/lymphoma 6
p53: Tumor protein p53
PRDM1: PR domain 1
c-Myc: v-myc avian myelocytomatosis viral oncogene homolog
PARP: Poly(ADP-ribose)polymerase
Caspase3: Cysteinyl aspartate protease
TNF: Tumor necrosis factor
TNFSF15: Tumor necrosis factor superfamily member 15
PAX-5: Paired box 5
PDCD2: Programmed cell death 2
Bcl-XL: B cell lymphoma x Large
STAT6: Signal transducer and activator of transcription 6
CIDE-3: Cell death inducing DFFA like effector c.
