# Supplementary material for: The feedback loop of LITAF and BCL6 is involved in regulating apoptosis in B cell non-Hodgkin's-lymphoma
Source: Oncotarget. 2016 Oct 15;7(47):77444–56. doi: 10.18632/oncotarget.12680 (PMC5363597; doi:10.18632/oncotarget.12680)
Supplement: Supplementary file 1 [file oncotarget-07-77444-s001.pdf]

The feedback loop of LITAF and BCL6 is involved in regulating apoptosis in B cell non-Hodgkin's-lymphoma

SUPPLEMENTARY TABLES

Supplementary Table S1: Association between expression of LITAF and BCL6 in 55 B-NHL cases

Variable	Cases	BCL6 protein		
		High expression	Low expression	*P* value
LITAF protein				0.0024
High expression	34	6	28	
Low expression	21	12	9	

Supplementary Table S2: Association between expression of BCL6 and active caspase3 in B-NHL cases

Variable	Cases	Active caspase3 protein		
		Positive	Negative	*P* value
BCL6 protein				0.0313
Positive	23	7	16	
Negative	17	11	6	

Supplementary Table S3: Summary of primer sequences used.

See Supplementary File 1
