# Supplementary material for: The feedback loop of LITAF and BCL6 is involved in regulating apoptosis in B cell non-Hodgkin's-lymphoma
Source: Oncotarget. 2016 Oct 15;7(47):77444–56. doi: 10.18632/oncotarget.12680 (PMC5363597; doi:10.18632/oncotarget.12680)
Supplement: Supplementary file 2 [file oncotarget-07-77444-s002.doc]

**Table S3** Summary of primer sequences used

| Name | Method | *5’* sequence | *3’* sequence |
| --- | --- | --- | --- |
| LITAF | qPCR | TCGGTTCCAGGACCTTACCA | ACGAAGGAGGATTCATGCCC |
| BCL6  PRDM1  c-Myc  GAPDH  Region A  Region B  Region C  BCL6-Luc  BCL6-Mut-A  BCL6-Mut-B  BCL6-Mut-C | qPCR  qPCR  qPCR  qPCR  CHIP  CHIP  CHIP  luciferase  luciferase  luciferase  luciferase | AGCAAGGCATTGGTGAAGACA  TCCAGCACT GTGAGGTTTCA  CGTCTCCACACATCAGCACAA  CAACGGATTTGGTCGTATTGG  CTAGTTGGAAAGAGCTGTGATG  AAAGAAGGAATTAGCCCCAGACC  GGCTTTGAGGGCTTTTGGTTGGCC  AAGGTACCAGCCACCACACCAGAAAGAA  GGTTCAAACCTCTCGCTAAATTTTGTGCATTTAGC  GGAAAGCAAAGCGCACTAAACCTCTTATGTCACCG  GCTGACACCAAGTCCTAAACTGCCACGTAGCAGTG | ATGGCGGGTGAACTGGATAC  TCAAACTCAGCCTCTGTCCA  CACTGTCCAACTTGACCCTCTTG  CTGGAAGATGGTGATGGGATT  CTTACCCATAAACTCAGCAGATC  GTGGGCTCCTCCTCTGTGACGTC  CTAAGAATCAACAGAGCTCAATTC  CCGCAAGCTTCGGCAGCAACAGCAATAATCACCT  GCTAAATGCACAAAATTTAGCGAGAGGTTTGAACC  CGGTGACATAAGAGGTTTAGTGCGCTTTGCTTTCC  CACTGCTACGTGGCAGTTTAGGACTTGGTGTCAGC |

Abbreviations: qPCR, quantitative polymerase chain reaction; GAPDH, glyceraldehyde-3-phosphate dehydrogenase; ChIP, chromatin immu- noprecipitation; Luc, luciferase; Region (A, B and C), three potential LITAF binding sequences within the promoter of BCL6; BCL6-Mut-(A, B and C), muted BCL6 luciferase reporters(CTCCC to CTAAA) in three different sites.
